# Supplementary material for: Characterization of BRCA1-deficient premalignant tissues and cancers identifies Plekha5 as a tumor metastasis suppressor
Source: Nat Commun. 2020 Sep 25;11:4875. doi: 10.1038/s41467-020-18637-9 (PMC7519681; doi:10.1038/s41467-020-18637-9)
Supplement: Supplementary file 1 — Supplementary Information [file 41467_2020_18637_MOESM1_ESM.pdf]

Supplementary Information

Characterization of *BRCA1*-deficient premalignant tissues and cancers identifies *Plekha5* as a tumor metastasis suppressor

Liu et al.

Supplementary Figure 1

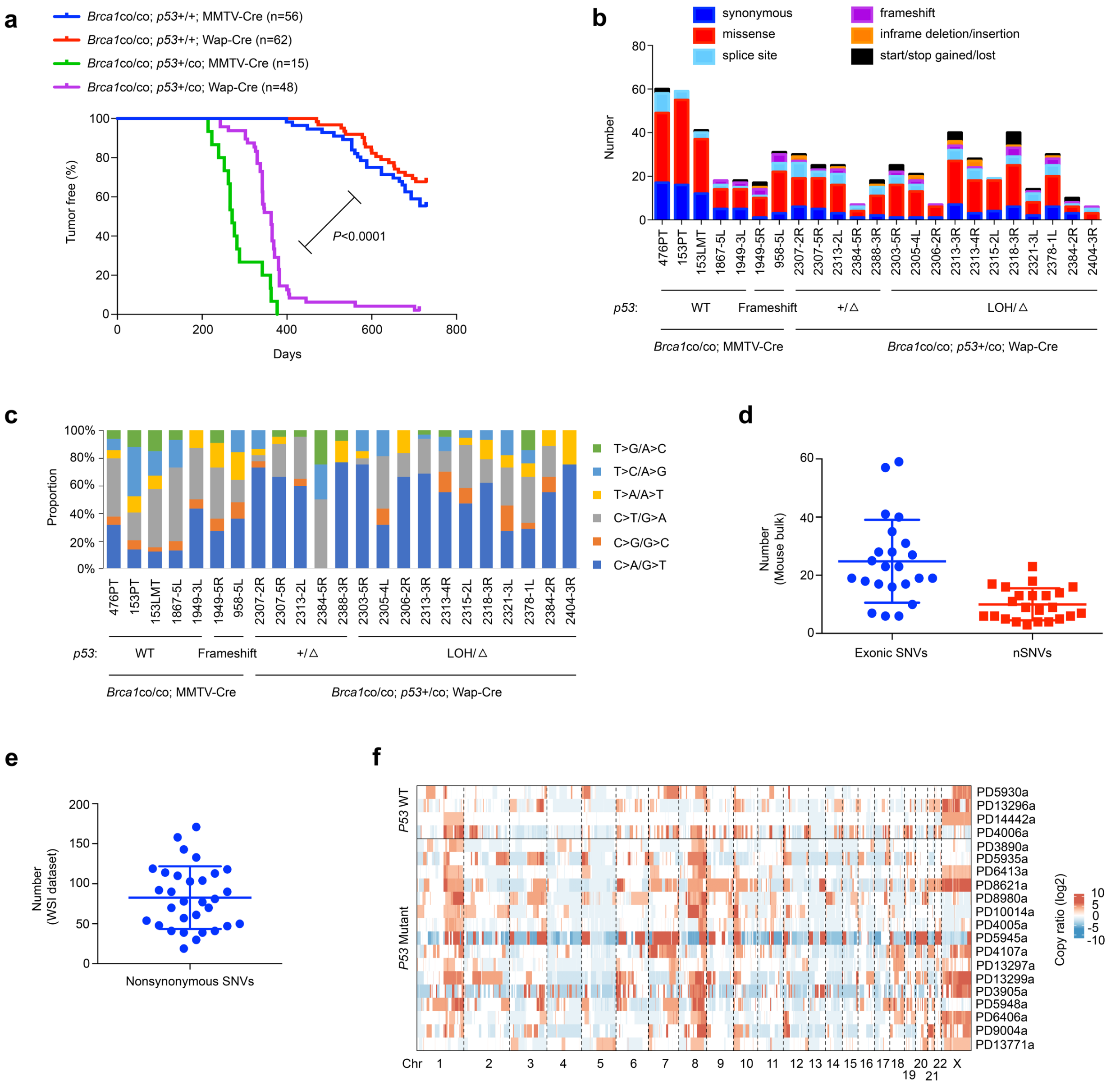

Supplementary Figure 1. Somatic mutations in *Brca1*-deficient breast tumors. **a** Tumor-free survival analysis of mice from *Brca1*$^{co/co}$;*p53*$^{+/+}$ *MMTV-* (n=56) or Wap- (n=62) Cre *and Brca1*$^{co/co}$;*p53*$^{+/co}$,*MMTV-* (n=15) or *Wap-* (n=48) *Cre* background. Significance determined by a two-sided the log-rank test (Mantel-Cox). **b** Number of exonic mutations according to the mutation type in *Brca1*$^{co/co}$;*p53*$^{+/+}$;*MMTV-Cre* and *Brca1*$^{co/co}$;*p53*$^{+/co}$,*WAP-Cre* mice. **c** Proportion of base pair change in each *Brca1*-deficient breast tumor of *Brca1*^{MKO} mice. **d** Number of all SNVs and nonsynonymous SNVs for human homologous genes in coding exon region in *Brca1*-deficient breast tumors of *Brca1*^{MKO} mice (n=23 tumors). **e** Number of nonsynonymous SNVs in breast tumors of patients with *BRCA1* germline mutations from the WSI dataset (n=31 tumors). **f** Copy-number variations (CNVs) profiles of breast tumors of patients with *BRCA1* germline mutations from the WSI dataset. Only the samples having CNV data were plotted,(n=20 tumors). The Integrative Genomics Viewer (IGV) was used for data visualization. Error bars represent SD.

Supplementary Figure 2

a

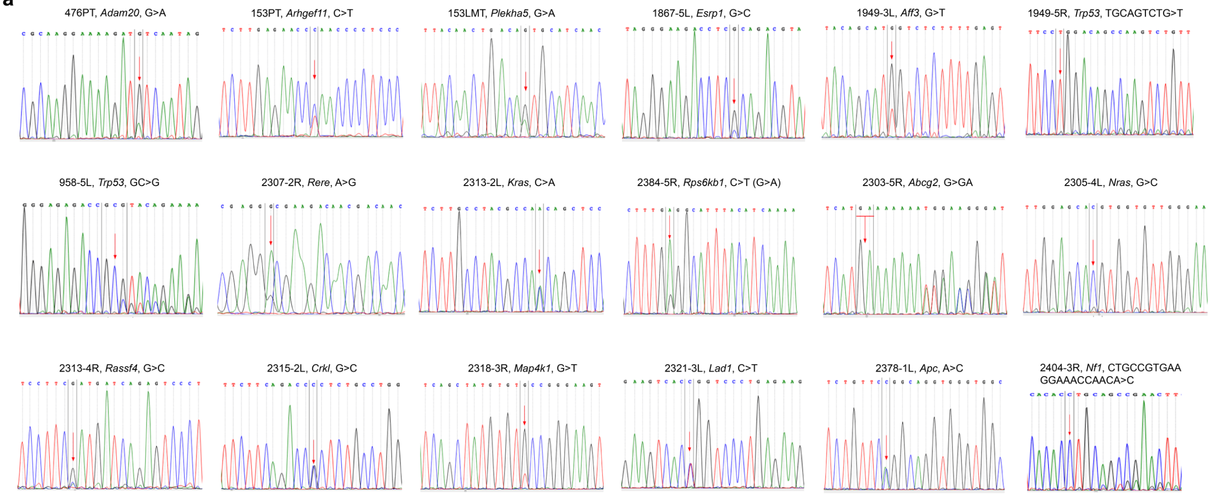

b

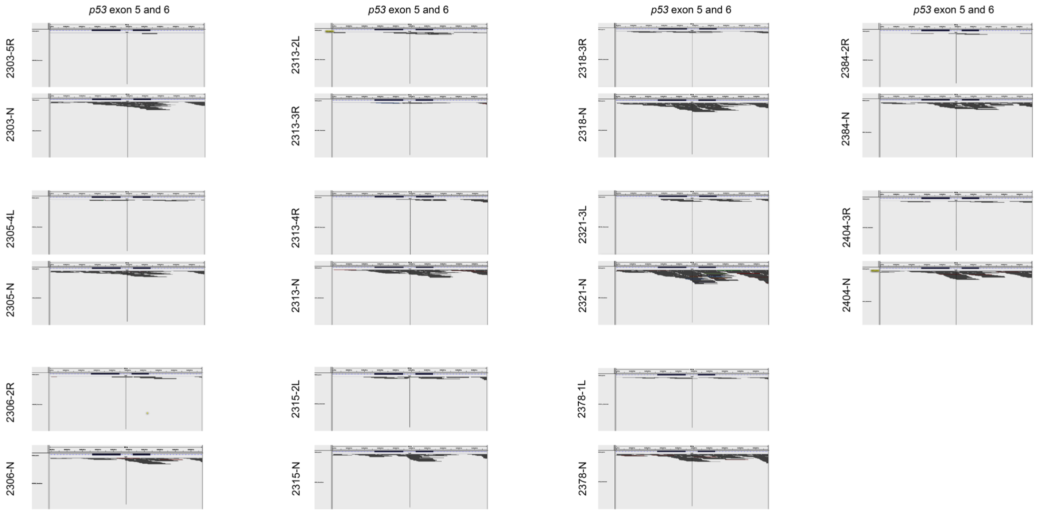

c

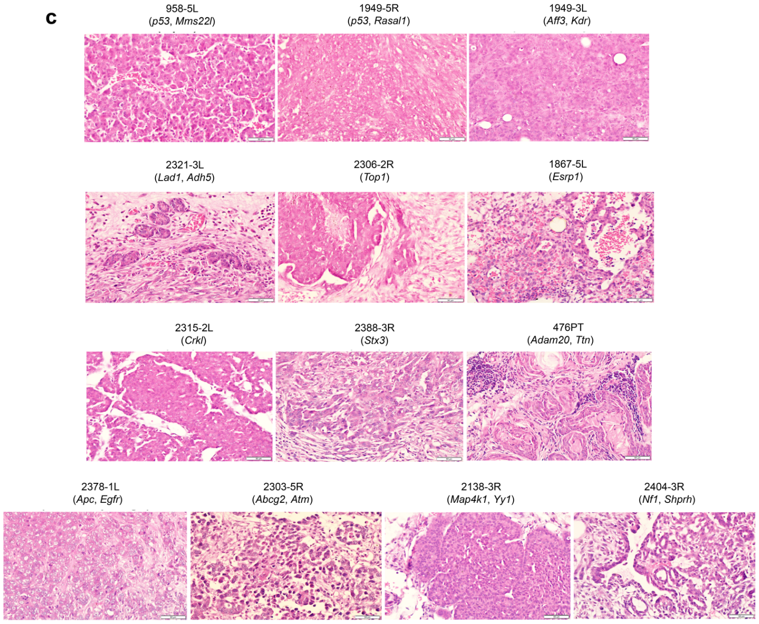

Supplementary Figure 2. Validation of driver mutations in *Brca1*-deficient breast tumors. **a** Validation of potential driver mutations for mouse tumors by Sanger sequencing. One potential driver mutation was selected for one tumor. **b** The Broad Institute Integrative Genomics Viewer (IGV) images showing the complete deletion (LOH) of exon 5-6 of *p53* in eleven tumors from mice with conditional heterozygous deletion of *p53* (+/△exon5-6) by reads of next-generation sequencing (NGS) on exon 5-6 of *p53*. Dark blue bar indicates the region of exon 5 and 6 of *p53*. N, normal. **c** Histology showing the morphology of tumors harboring different driver mutations. One representative section was shown for each tumor. Scale bars, 50 μm.

Supplementary Figure 3

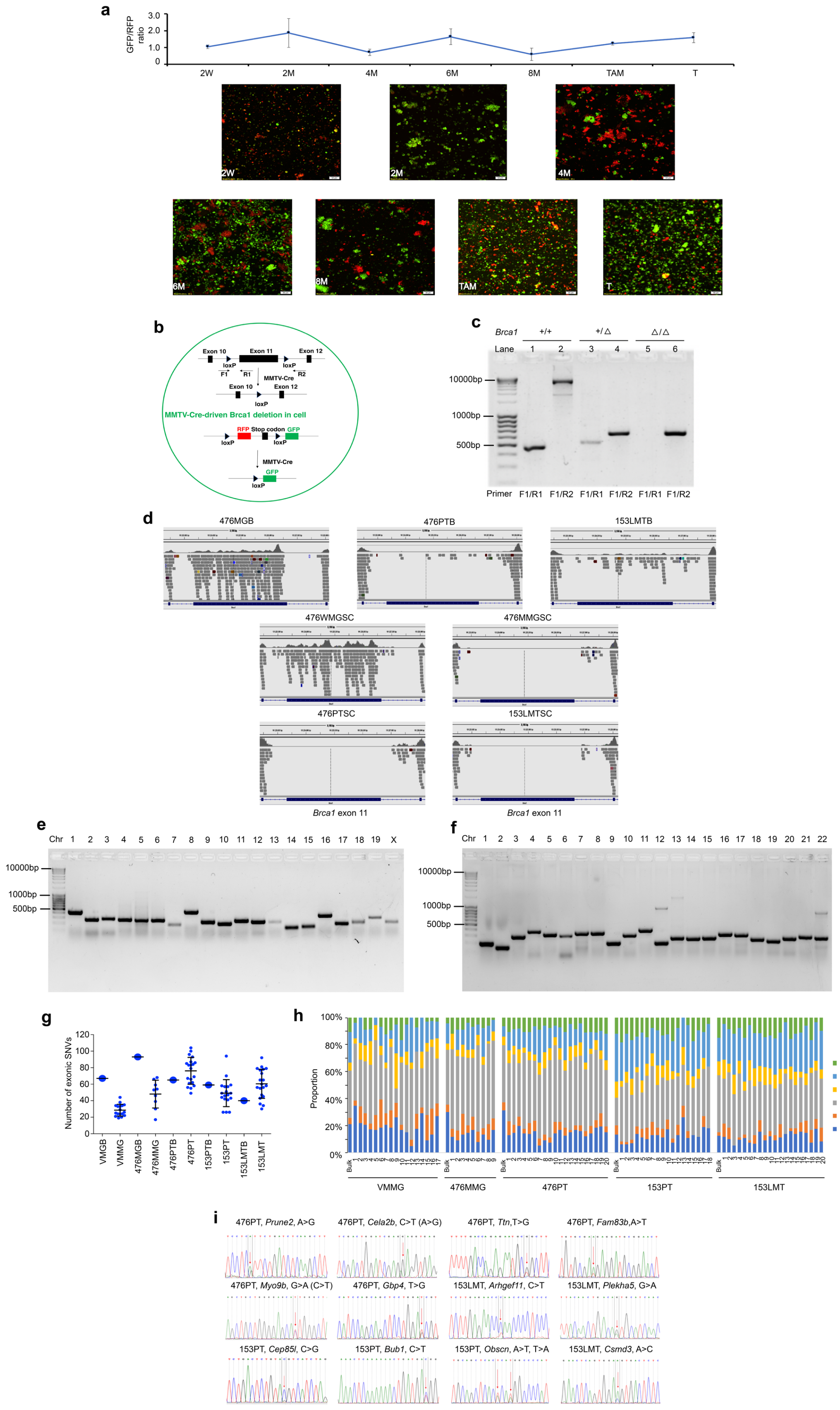

Supplementary Figure 3. scWES of premalignant *Brca1*-deficient mammary gland cells and tumor cells. **a** *Brca1*-MT cells/*Brca1*-WT cells ratio (GFP/RFP ratio) in *Brca1*-deficient mammary gland and tumor from mice of different ages (n=3 images). Tomato (RFP) indicates *Brca1*-WT cell, GFP indicates *Brca1*-MT cell. WT, wild type; MT, mutant; W, week; M, month; TAM: tumor-adjacent mammary gland; T: tumor; RFP: red fluorescent protein. Scale bar, 50μm. **b** Reporter structure of *Brca1*$^{co/co}$*;MMTV-Cre*;*mT/mG* mice. **c** Representative agarose gel image showing *Brca1* genotype of single cells from *Brca1*$^{co/co}$*;MMTV-Cre;mT/mG* mice using two pairs of primer. F1/R1 (as shown in **b**) for *Brca1*-WT cell, size of the product is 470bp; F1/R2 (as shown in **b**) for *Brca1* mutant cell (exon 11 deletion), size of the product is 621bp. '+/+' indicates *Brca1* WT (two copies of DNA with exon 11), '+/Δ' indicates *Brca1* heterozygous mutation (only one copy of the DNA with exon 11), 'Δ/Δ' indicates *Brca1* homozygous mutation (two copies of DNA without exon 11). Lanes 1 and 2 were loaded with DNA of *Brca1*-WT cells. Lanes 3 and 4 were loaded with DNA of cells carrying *Brca*1 heterozygous mutation. Lanes 5 and 6 were loaded with DNA of cells carrying *Brca*1 homozygous mutation. The cells with '+/Δ' were excluded from our study. **d** The IGV images showing genotype of *Brca*1 by reads of NGS on exon 11 of *Brca1*. Blue bar indicates the region of exon 11 of *Brca1*. B, Bulk; SC, single cell; WMG: wild-type mammary gland. **e** Representative agarose gel image showing the efficiency of whole genome amplification of single cells from mice. **f** Representative agarose gel image showing the efficiency of whole genome amplification of single cells from the PDX models. **g** Number of exonic SNVs for different tumor developmental stages of single cells and paired bulk samples. A big dot indicates a bulk sample, a small dot indicates a single cell (VMGB, n=1 bulk tissue; VMMG, n=17 cells; 476MGB, n=1 bulk tissue; 476MMG, n=9 cells; 476PTB, n=1 bulk tissue; 476PT, n=20 cells; 153PTB, n=1 bulk tissue; 153PT, n=18 cells; 153LMTB, n=1 bulk tissue; 153LMT, n=20 cells). **h** Stacked bar plot depicting the proportion of six different types of base pair change in different tumor developmental stages of *Brca1*-deficient single cells and bulk. **i** Sanger sequencing validating the SNVs calling by scWES. Error bars represent SD.

Supplementary Figure 4

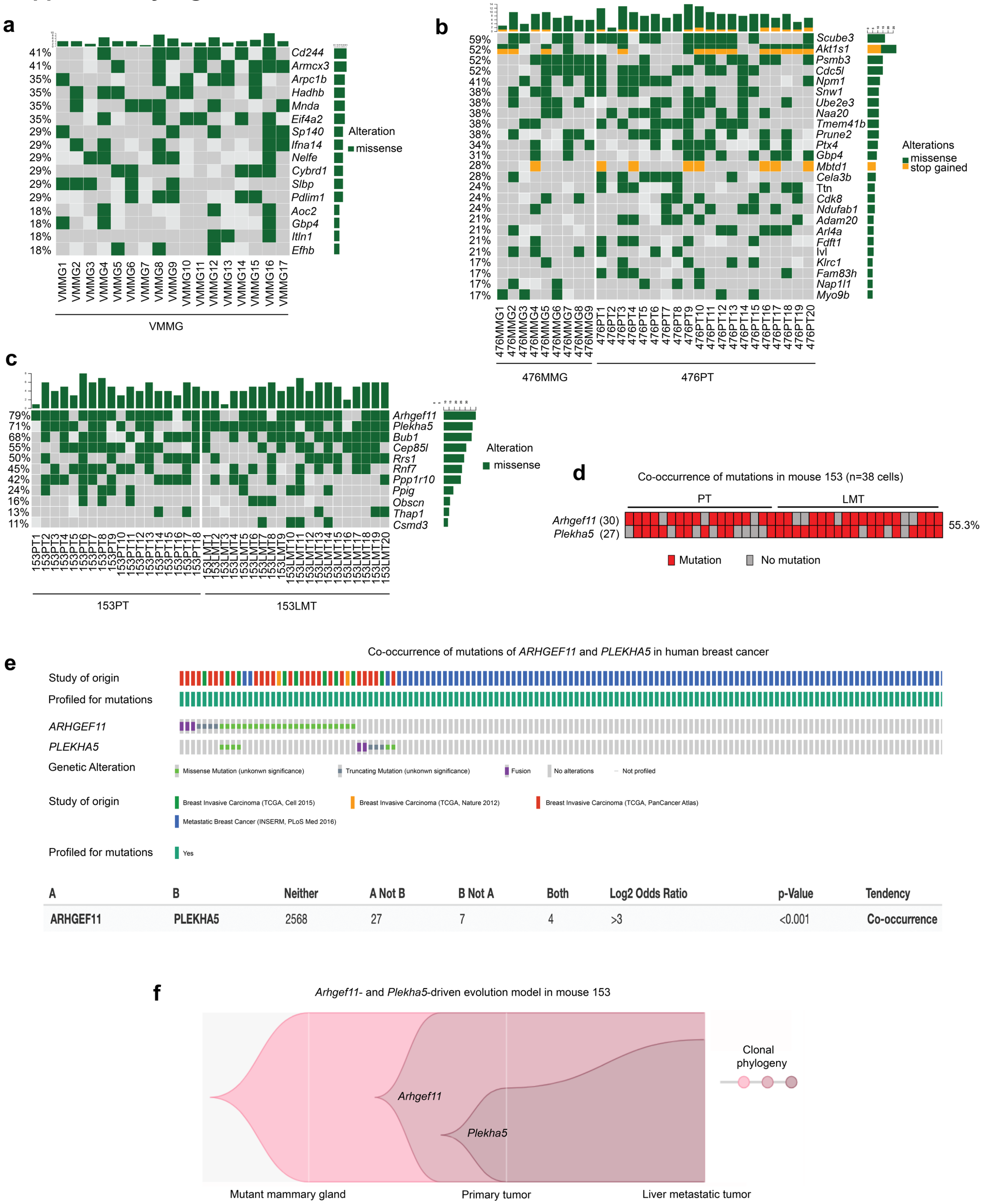

Supplementary Figure 4. Somatic SNVs of single cells in each mouse. **a-c** Oncoplots summarizing recurrently mutated genes in VMMG (**a**), 476MMG and 476PT (**b**), 153PT, and 153LMT (**c**) single cells. A grey rectangle indicates the wild-type status of the indicated site, a light grey rectangle indicates no coverage of the indicated site. **d** Co-occurrence of *Arhgef11* and *Plekha5* mutation in PT and LMT of mouse 153 (n=38 cells, red rectangle indicates mutation, grey rectangle indicates no mutation or no coverage). **e** Co-occurrence of *ARHGEF11* and *PLEKHA5* mutation in human breast cancer from the TCGA and INSERM datasets analyzed by using cBioportal. N=2606 cases. **f** Evolution model of mouse 153 based on putative driver genes.

Supplementary Figure 5

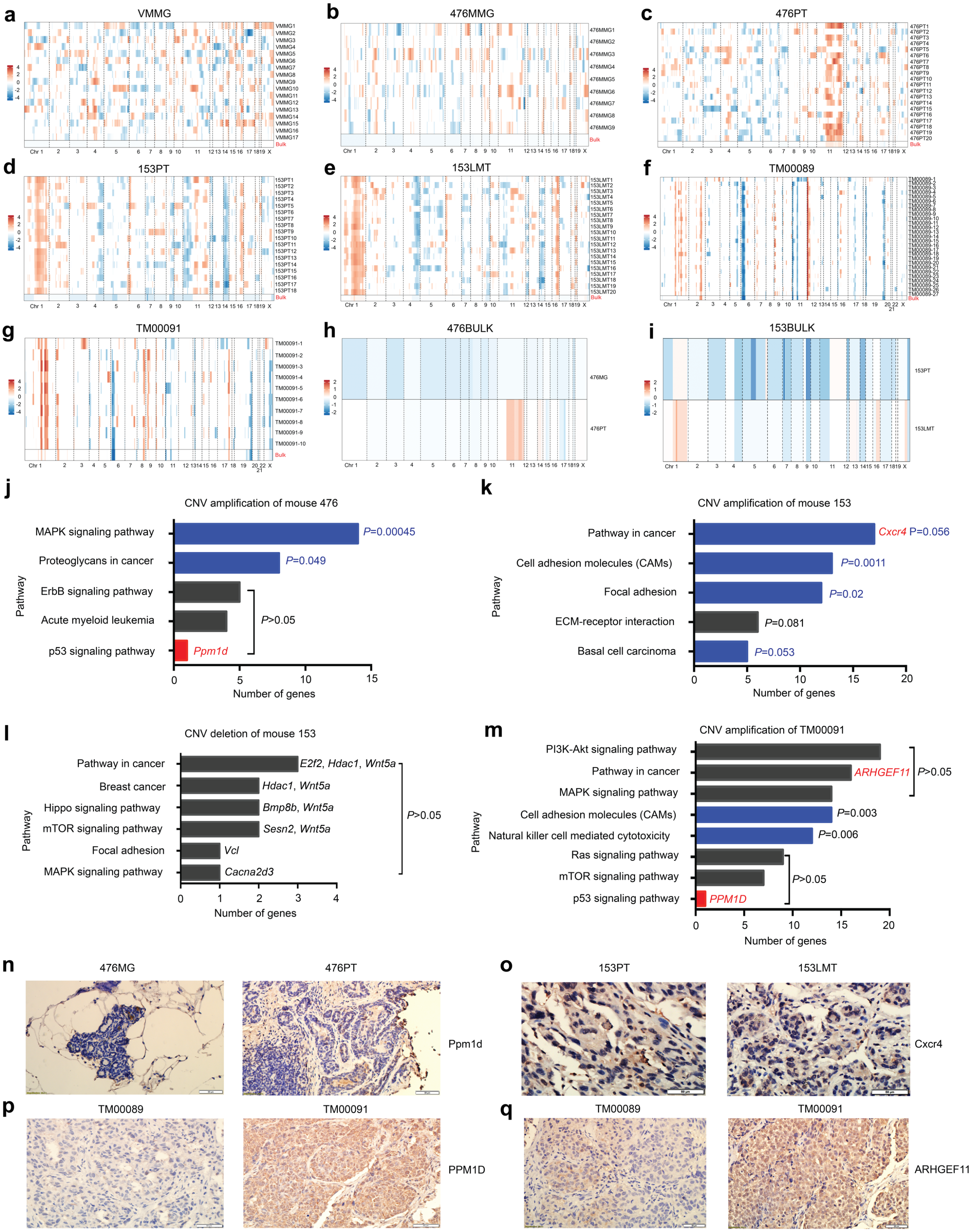

Supplementary Figure 5. Validation of CNV calling of scWES and pathway analysis for CNV candidates. **a-g** Inferred CNV profiles of single cells (top) and paired bulk (bottom) by WES in (**a**) virgin mouse, (**b, c**) mouse 476, (**d, e**) mouse 153, (**f**)TM00089, (**g**)TM00091. **h-i** Inferred CNV profiles of bulk samples for (**h**) mouse 476 (476MGB, 476PTB) and (**i**) mouse 153 (153PTB, 153LMTB). **j-m** DAVID-KEGG pathway analysis of genes with amplification in single cells of mouse 476 (chromosome 11) (**j**), genes with amplification in single cells of mouse 153 (chromosome 1, 6, 16) (**k**), genes with deletion in single cells of mouse 153 (chromosome 4, 14) (**l**), genes with amplification (chromosome 1, 17) in single cells of PDX model TM00091 (**m**). The blue emphasizes the *P* vale is <0.05 in the DAVID-KEGG pathway analysis, the grey indicates the *P* vale is >0.05, and the red emphasizes the driver genes we describe in detail in the main text. A modified Fisher's exact test was used for enrichment *P* value calculation in DAVID-KEGG functional annotation analysis. **n** IHC staining of Ppm1d in 476MG and 476PT tissue. Scale bar, 50μm. **o** IHC staining of Cxcr4 in 153PT and 153LMT tissue. Scale bar, 50μm. **p-q** IHC staining of PPM1D (**p**) and ARHGEF11 (**q**) in TM00089 and TM00091 tissue. Scale bar, 50μm.

Supplementary Figure 6

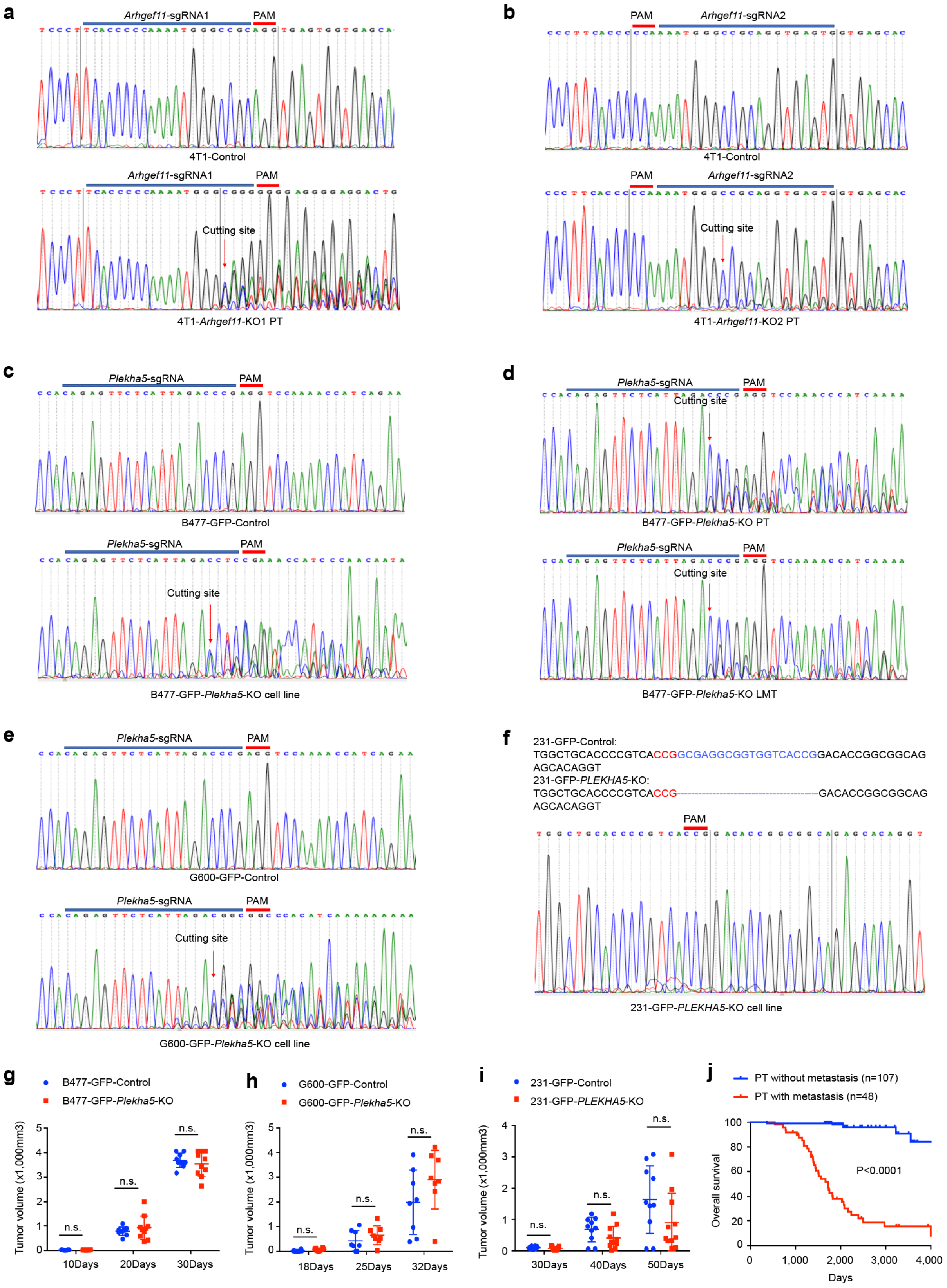

Supplementary Figure 6. Validation of *Arhgef11* and *Plekha5* knockout and tumor growth. **a-b** Sanger sequencing for the primary tumors from BALB/c mice implanted with 4T1 control cells (top) and 4T1-*Arhgef11*-KO1 cells (bottom) (**a**), 4T1 control cells (top) and 4T1-*Arhgef11*-KO2 cells (bottom) (**b**) **c-f** Sanger sequencing for B477-GFP control cell (top) and *Plekha5*-KO B477-GFP cell (bottom)(**c**), the primary tumor (top) and liver metastatic tumor (bottom) grow from nude mice implanted with *Plekha5*-KO B477-GFP cells (**d**), G600-GFP control cell (top) and *Plekha5*-KO G600-GFP cell (bottom)(**e**), *PLEKHA5*-KO MDA-MB-231-GFP cells (**f**). **g-i** Measurement of tumor volume after implantation of B477-GFP-Control and B477-GFP-*Plekha5*-KO cell (**g**), G600-GFP-Control and G600-GFP-*Plekha5*-KO cell (**h**), MDA-MB-231-GFP-Control and MDA-MB-231-GFP-*PLEKHA5*-KO cell (**i**) into mammary fat pat ($5X10^5$ tumor cells per injection, n=8-11 mice per group). Data were reported as the mean ± SD. Significance determined by two-tailed Student's *t*-test. n.s., not significant. **j** Kaplan-Meier curves showing the correlation between patients with or without metastasis and clinical outcome, as analyzed for overall survival. Significance determined by a two-sided log-rank test (Mantel-Cox).
