# Supplementary material for: Characterization of BRCA1-deficient premalignant tissues and cancers identifies Plekha5 as a tumor metastasis suppressor
Source: Nat Commun. 2020 Sep 25;11:4875. doi: 10.1038/s41467-020-18637-9 (PMC7519681; doi:10.1038/s41467-020-18637-9)
Supplement: Supplementary file 3 — Description of Additional Supplementary Files [file 41467_2020_18637_MOESM3_ESM.pdf]

Description of Additional Supplementary Files

File name: Supplementary Data 1
Description: Summary of bulk and single-cell samples and sequencing data.

File name: Supplementary Data 2
Description: Mutations in mouse bulk samples.

File name: Supplementary Data 3
Description: CNVs in bulk and single cells.

File name: Supplementary Data 4
Description: Primers for PCR-Sanger sequencing validation of SNVs.

File name: Supplementary Data 5
Description: Mutations in single cells.

File name: Supplementary Data 6
Description: Probes and primers for ddPCR.
