# Supplementary material for: Characterization of BRCA1-deficient premalignant tissues and cancers identifies Plekha5 as a tumor metastasis suppressor
Source: Nat Commun. 2020 Sep 25;11:4875. doi: 10.1038/s41467-020-18637-9 (PMC7519681; doi:10.1038/s41467-020-18637-9)
Supplement: Supplementary file 10 — Reporting Summary [file 41467_2020_18637_MOESM10_ESM.pdf]

nature research

Corresponding author(s): Xiaoling Xu

Last updated by author(s): Aug 6, 2020

Reporting Summary

Nature Research wishes to improve the reproducibility of the work that we publish. This form provides structure for consistency and transparency in reporting. For further information on Nature Research policies, see our Editorial Policies and the Editorial Policy Checklist.

Statistics

For all statistical analyses, confirm that the following items are present in the figure legend, table legend, main text, or Methods section.

n/a	Confirmed	
☐	☒	The exact sample size (n) for each experimental group/condition, given as a discrete number and unit of measurement
☐	☒	A statement on whether measurements were taken from distinct samples or whether the same sample was measured repeatedly
☐	☒	The statistical test(s) used AND whether they are one- or two-sided<br>*Only common tests should be described solely by name; describe more complex techniques in the Methods section.*
☒	☐	A description of all covariates tested
☒	☐	A description of any assumptions or corrections, such as tests of normality and adjustment for multiple comparisons
☐	☒	A full description of the statistical parameters including central tendency (e.g. means) or other basic estimates (e.g. regression coefficient) AND variation (e.g. standard deviation) or associated estimates of uncertainty (e.g. confidence intervals)
☐	☒	For null hypothesis testing, the test statistic (e.g. F, t, r) with confidence intervals, effect sizes, degrees of freedom and P value noted<br>*Give P values as exact values whenever suitable.*
☒	☐	For Bayesian analysis, information on the choice of priors and Markov chain Monte Carlo settings
☒	☐	For hierarchical and complex designs, identification of the appropriate level for tests and full reporting of outcomes
☒	☐	Estimates of effect sizes (e.g. Cohen's d, Pearson's r), indicating how they were calculated

Our web collection on statistics for biologists contains articles on many of the points above.

Software and code

Policy information about availability of computer code

Data collection	For data collection the following software was used: Image Lab version 6.0.1(Bio-Rad), QuantaSoft software 1.7.4 (BioRad), R 3.5.1, R package GEOquery 2.56.0.
Data analysis	CASAVA 1.8.1 pipeline (Illumina Inc.) was used to process NGS data; BWA-MEM algorithm from the Burrows-Wheeler Aligner software 0.7.12-r1039 was used to do sequence alignment; PICARD 2.5.0 was used to mark the duplicate reads; GATK 3.6 IndelRealigner was used to do realignment; GATK 3.6 BaseRecalibrator was used to recalibrate the base quality scores; MuTect2 was used to call somatic SNVs in bulk samples; SAMtools 1.3 and VarScan 2.3.9 were used to call somatic SNVs in single cells and their matched bulk tissue; CNVkit 0.8.5 was used to call CNVs in bulk samples; A RPKM method was used to estimate CNVs in single cells, the code was deposited in Github; R package ComplexHeatmap 2.0.0 was used to summarized and plotted SNV data; R package deconstructSigs 1.8.0 was used to generate mutational signature; R package phangorn 2.5.5 was used to generate phylogenetic tree; R package TimeScape 1.8.0 was used to generate clone lineage; Unipro UGENE 1.30.0 was used for sequence analysis; QuantaSoft software 1.7.4 (BioRad) was used for analysis of the ddPCR data; Image J 1.51 was used for counting crystal violet in migration and invasion assays and GFP signals of organs with metastases; GraphPad Prism 6 was used for generating all bar plots, box plots, dot plots, line chart, Kaplan-Meier curves. Calculations of statistical significance were performed using GraphPad Prism 6.0. R 3.5.1 was used for data analysis and all R code is available at: https://github.com/Radhav-XuLab/JianlinLiuSingleCell.

For manuscripts utilizing custom algorithms or software that are central to the research but not yet described in published literature, software must be made available to editors and reviewers. We strongly encourage code deposition in a community repository (e.g. GitHub). See the Nature Research guidelines for submitting code & software for further information.

Data

Policy information about availability of data

All manuscripts must include a data availability statement. This statement should provide the following information, where applicable:

- Accession codes, unique identifiers, or web links for publicly available datasets
- A list of figures that have associated raw data
- A description of any restrictions on data availability

The NGS raw data that support the findings of this study are available in the Sequence Read Archive (SRA), National Center for Biotechnology Information (NCBI), under accession number SUB7750623 (PDX single cells), SUB7767489 (PDX bulk tissues), SUB7699323 (Mouse single cells), and SUB7768225 (Mouse bulk tissues). The information of samples and the NGS data is provided in Supplementary Table 1. Somatic mutations are provided in Supplementary Table 2 and 5. CNVs are provided in Supplementary Table 3. Sanger sequencing results and primers for SNVs are provided in Supplementary Table 4. The details of the pathway analysis of genes with CNVs in single cells are provided in Supplementary Table 6. Primers and probes for ddPCR are provided in Supplementary Table 7. The SNV and CNV data of the patients with BRCA1 germline mutations of WSI dataset21 are downloaded from the ICGC (https://icgc.org). Microarray gene expression data including clinical outcomes of 155 breast cancer patients bearing primary tumors with or without metastasis were retrieved from Gene Expression Omnibus (GEO) under accession number GSE9893 (https://www.ncbi.nlm.nih.gov/geo/query/acc.cgi?acc=GSE9893)57. The source data underlying Figures 1a, b, 2a, b, 3e, f, 4a, c-e, 5e-i, 6c, e, g, i, k, l and Supplementary Figures 1a-f, 3c, e-h, 5j-m, 6g-l are provided as Source Data file. All other data and materials can be requested from the corresponding author upon reasonable request.

Field-specific reporting

Please select the one below that is the best fit for your research. If you are not sure, read the appropriate sections before making your selection.

☒ Life sciences ☐ Behavioural & social sciences ☐ Ecological, evolutionary & environmental sciences

For a reference copy of the document with all sections, see nature.com/documents/nr-reporting-summary-flat.pdf

Life sciences study design

All studies must disclose on these points even when the disclosure is negative.

Sample size	No sample size calculation was performed, sample sizes were chosen based on previous experience and on what is common practice in the field.
Data exclusions	No data were exclude from the analyses.
Replication	All experiments were independently repeated as indicated and were reliably reproduced (see Figure Legend where applicable for further details). Multiple cell lines were used to confirm reproducibility of the findings.
Randomization	Nude mice or BABL/c mice were at the same age and were random allocated into control and experiment group. Randomization was not relevant to other experiments, as they were in-vitro.
Blinding	Blinding was not performed during experiments to facilitate staff monitoring.

Reporting for specific materials, systems and methods

We require information from authors about some types of materials, experimental systems and methods used in many studies. Here, indicate whether each material, system or method listed is relevant to your study. If you are not sure if a list item applies to your research, read the appropriate section before selecting a response.

Materials & experimental systems

n/a	Involved in the study	
☐	☒	Antibodies
☐	☒	Eukaryotic cell lines
☒	☐	Palaeontology and archaeology
☐	☒	Animals and other organisms
☒	☐	Human research participants
☒	☐	Clinical data
☒	☐	Dual use research of concern

Methods

n/a	Involved in the study	
☒	☐	ChIP-seq
☒	☐	Flow cytometry
☒	☐	MRI-based neuroimaging

Antibodies

Antibodies used	Kras (Abcam, catalog #ab180772, Rabbit polyclone, lot #GR3264774-1), Nras (Abcam, catalog #ab206969, Rabbit EPR18713-13, lot #GR20284-1), Tpr (Abcam, catalog #ab84516, Rabbit polyclone, lot #GR200164-29), Akt3 (Abcam, catalog #ab152157, Rabbit

	polyclone, lot #GR119291-35), Vimentin (Abcam, catalog #ab92547, Rabbit EPR3776, lot # GR47955-1), Hepatocyte (HepPar1) (Dako, catalog #M7158, Mouse OCH1E5, lot #00047868), Ppm1d (Abcam, catalog #ab31270, Rabbit polyclone, lot #GR3228201-1), Cxcr4 (Abcam, catalog #ab124824, Rabbit UMB2, lot #GR3190294-6), Arhgef11 (Santa Cruz Biotechnology, catalog #sc-166740, Mouse D-9, lot #12110), Plekha5 (Santa Cruz Biotechnology, catalog #sc-390311, Mouse E-2, lot #D0615), CK14 (Abcam, catalog #49806, Mouse LL002, lot #GR112506-2), goat anti-mouse IgG secondary antibody (Invitrogen, catalog#A32727, polyclone)
Validation	Kras (#ab180772, Host species: Rabbit; Species reactivity: Mouse, Rat, Human; Tested applications: WB, IHC-P, IP, ICC/IF), Nras (#ab206969, Host species: Rabbit; Species reactivity: Mouse, Rat, Human; Tested applications: WB, IHC-P, IP, ICC/IF, Flow cyt), Tpr (#ab84516, Host species: Rabbit; Species reactivity: Mouse, Human; Tested applications: IHC-P, ICC/IF), Akt3 (#ab152157, Host species: Rabbit; Species reactivity: Mouse, Human; Tested applications: IHC-P, WB, ICC/IF), Vimentin (#ab92547, Host species: Rabbit; Species reactivity: Mouse, Rat, Human, Rhesus monkey; Tested applications: IHC-P, IHC-Fr, WB, ICC/IF, Flow Cyt), Hepatocyte (HepPar1) (#M7158, Host species: Mouse; Species reactivity: Mouse, Human, Cat; Tested applications: IHC), Ppm1d (#ab31270, Host species: Rabbit; Species reactivity: Mouse, Rat, Xenopus laevis, Zebrafish; Tested applications: IHC-P, WB), Cxcr4 (#ab124824, Host species: Rabbit; Species reactivity: Mouse, Rat, Human; Tested applications: IHC-P, IHC-Fr, WB, ICC/IF, Flow Cyt), Arhgef11 (#sc-166740, Host species: Mouse; Species reactivity: Mouse, Rat, Human; Tested applications: IHC-P, WB, IP, ICC/IF, ELISA), Plekha5 (#sc-390311, Host species: Mouse; Species reactivity: Mouse, Rat, Human; Tested applications: IHC-P, WB, IP, ICC/IF, ELISA), CK14 (#49806, Host species: Mouse; Species reactivity: Mouse, Rat, Human; Tested applications: IHC-P, WB, ICC/IF), goat anti-mouse IgG secondary antibody (#A32727, Host species: Goat; Species reactivity: Mouse; Tested applications: ICC/IF; WB)

Eukaryotic cell lines

Policy information about cell lines

Cell line source(s)	The immortalized mouse Brca1-WT epithelial cell line B477 was derived from the mammary gland of Brca1-WT mice (Brca+/+; p53+/-). The mouse Brca1-deficient epithelial cell line G600 was derived from the mammary gland of Brca1-deficient mice (Brca1△exon11/△exon11; p53+/-). The 4T1 cell line, MDA-MB-231 cell line, and the 293FT cell line were obtained from American Tissue Culture Collection (ATCC).
Authentication	Morphology check by microscope was done for each cell line for authentication.
Mycoplasma contamination	All cell lines tested negative for mycoplasma contamination.
Commonly misidentified lines<br>(See ICLAC register)	None.

Animals and other organisms

Policy information about studies involving animals; ARRIVE guidelines recommended for reporting animal research

Laboratory animals	Mixed background of FVB/129SvEv/Black Swiss female mice of 2 weeks, 2 months, 4 months, 6 months, 8 months, 11 months; BALB/c and nude female mice of 6-8 weeks.
Wild animals	The study did not involve wild animals.
Field-collected samples	The study did not involve samples collected from the field.
Ethics oversight	Faculty of Health Sciences, University of Macau. Protocol Name: Request for amendment of approved animal research ethics application. Protocol ID: UMARE-AMEND-100.

Note that full information on the approval of the study protocol must also be provided in the manuscript.
